# Prevalence of urinary tract infection and antimicrobial resistance patterns of uropathogens with biofilm forming capacity among outpatients in morogoro, Tanzania: a cross-sectional study

**DOI:** 10.1186/s12879-023-08641-x

**Published:** 2023-10-05

**Authors:** Eulambius M. Mlugu, Juma A. Mohamedi, Raphael Z. Sangeda, Kennedy D. Mwambete

**Affiliations:** 1 Department of Pharmaceutics and Pharmacy Practice, School of Pharmacy, Muhimbili University of Health and Allied Sciences, Dar es Salaam, Tanzania; 2 Department of Pharmaceutical Microbiology, School of Pharmacy, Muhimbili University of Health and Allied Sciences, Dar es Salaam, Tanzania

**Keywords:** UTI, Antimicrobial resistance, Biofilm forming bacteria

## Abstract

**Introduction:**

Urinary tract infection (UTI) is the second most common infectious disease affecting more than 150 million people globally annually. Uropathogenic *E. coli* (UPEC), the predominant cause of UTI, can occur as a biofilm associated with antimicrobial resistance (AMR). There is a data gap on global AMR patterns from low-income settings, including Tanzania. Data on antimicrobial susceptibility patterns in relation to biofilm formation will help in the proper selection of antibiotics and the fight against AMR.

**Methods:**

This analytical cross-sectional study was conducted among consecutively selected outpatients (n = 344) from January to May 2022 at Morogoro Regional Referal Hospital. Mid-stream urine samples were collected aseptically from symptomatic patients. A significant UTI was defined when more than 10^5^ colonies/ml of urine were recorded. Kirby Bauer’s disc diffusion method was used for antibiotics susceptibility patterns and a Congo Red Agar method was used to determine biofilm formation. Two-sided χ2 test or Fisher’s exact test, Cohen’s kappa coefficient and logistic regression were used for data analysis. A p-value < 0.05 was considered statistically significant.

**Results:**

The prevalence of UTIs was 41% (141/344) and elders (>=60 years) had five times higher odds of having UTI as compared to adolescents (p < 0.001). *E. coli* was the most predominant bacteria (47%; 66/141), which displayed moderate susceptibility against ciprofloxacin (59.1%) and nitrofurantoin (57.6%). A total of 72 (51%) of all isolated bacteria were multi-drug resistant. All isolated bacteria demonstrated high resistance (> 85%) against ampicillin and co-trimoxazole. In this study, 51.5% (34/66) were biofilm-forming *E. coli* and demonstrated relatively higher antibiotic resistance as compared to non-biofilm forming bacteria (p < 0.05).

**Conclusion:**

We report high antibiotic resistance against commonly used antibiotics. Slightly more than half of the isolated bacteria were biofilm forming *E. coli.* A need to strengthen stewardship programs is urgently advocated.

## Background

Urinary tract infection (UTI) is a disease of public health importance affecting more than 150 million people [1] with a financial burden of about $6 billion worldwide each year [2]. It is one of the most common infectious diseases, second to upper respiratory tract infections [3]. More than 50% of all women and at least 12% of men experience UTI in their lifetime [4]. Data regarding the prevalence of UTI among children and pregnant women are largely available [5–7], probably because of their susceptibility to secondary complications. Nevertheless, data on UTI prevalence in the general population are scanty in Tanzania indicating the need to regularly monitor the burden of UTIs to inform policy decisions.

For the majority of UTIs, bacteria from the Enterobacteriaceae family are the most frequent culprits. Uropathogenic *Escherichia coli* (UPEC) are the most prevalent strains causing UTIs, accounting for about 80% of uncomplicated UTIs and about 95% of community and hospital-acquired infections [8, 9]. Bacteria colonize the urinary tract by either freely attaching to the epithelial surface (planktonic) reversibly [2], or forming biofilm [10] defined by the presence of bacterial aggregates embedded in a self-produced extracellular matrix [11].

The human immune system has trouble recognizing bacteria and getting rid of infections with biofilm. Additionally, antibiotics penetration to the deeper layers of the biofilm matrix is difficult and may expose bacteria to sub-therapeutic antimicrobial concentrations leading to resistance [12]. Consequently, biofilm-associated infections tolerate the standard 5–10-day antibiotics treatment [13, 14], indicating the need for multi-targeted or combination antibiotics therapies. Availability of data on the biofilm forming patterns in relation to antimicrobial resistance (AMR) may guide the designing of appropriate antibiotics policy guidelines and, eventually, the prevention of AMR. Few studies have reported the relationship between biofilm forming and AMR [15–17]. However, such data are scarce from Sub-Saharan Africa and Tanzania included [18, 19].

Data regarding antimicrobial susceptibility patterns are reported [20]. However, the patterns of AMR differ across geographical locations and there is data gap in many low-income settings [21]. The present study investigated the prevalence of UTI in the general population, biofilm forming ability of UPEC and AMR in clinical isolates at Morogoro referral regional hospital, Tanzania.

## Methods

### Study design and setting

**Fig. 1:**
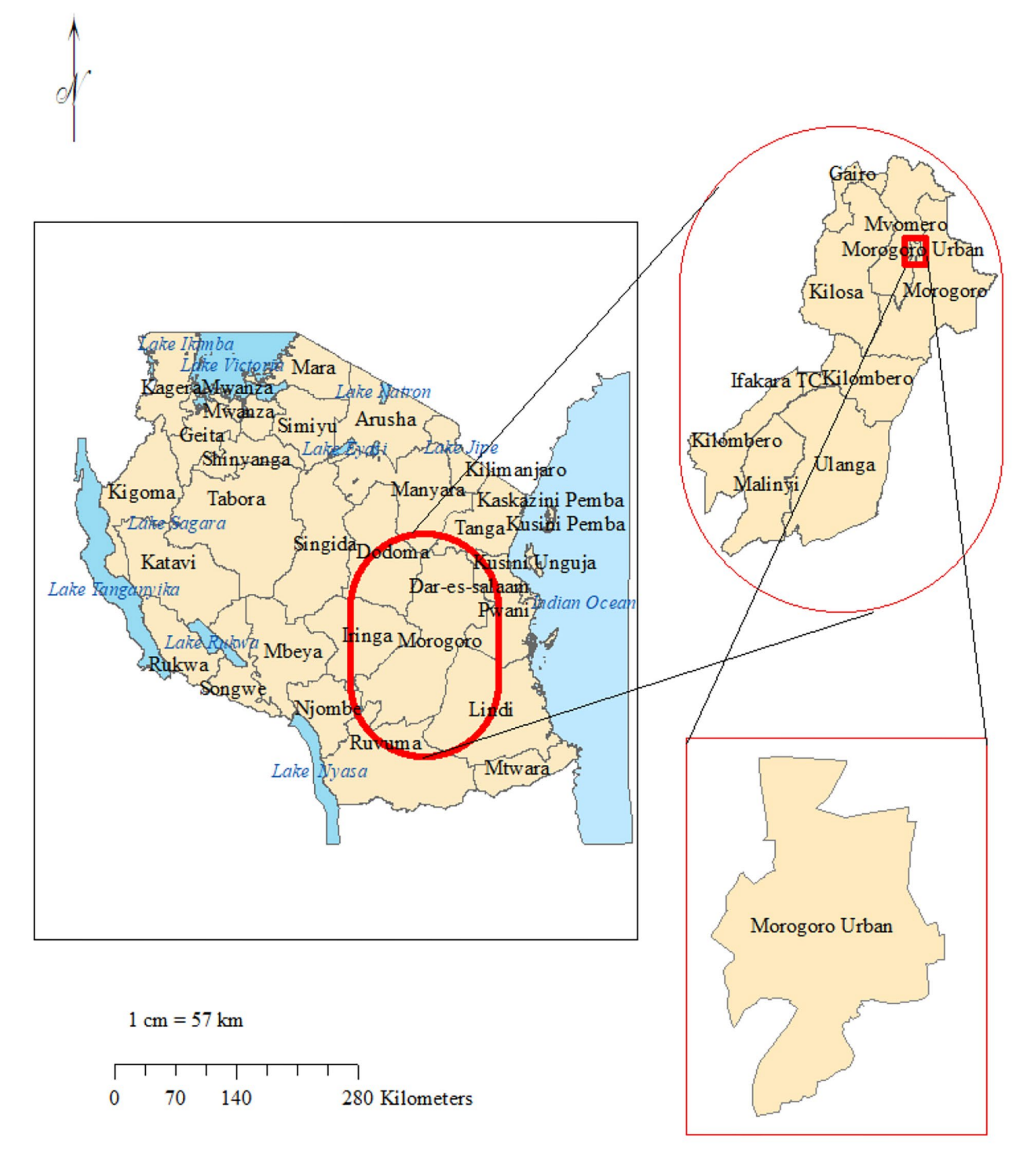
Study site map. The top right is the map of the Morogoro region. Bottom right is the map of Morogoro Urban. On the left is the map of Tanzania. The study site map was originally generated using ArcGIS software version 10.7.1 (Esri, California, USA; https://www.esri.uconn.edu/software/arcgis-student/)

This was an analytical cross-sectional study conducted at Morogoro referral regional hospital (MRRH). MRRH is located in the Morogoro region, about 190 km west of Dar es Salaam, the largest business city in Tanzania (Fig. 1). The study site choice was motivated by the scarcity of data regarding AMR patterns from the region, which is required for antimicrobial stewardship activities.

### Patient recruitment and data collection

The study targeted all patients attending outpatient clinics with any symptoms of dysuria, urination frequency, urgency, fever, hematuria and suprapubic pain. A standard pilot-tested questionnaire was used to collect patients’ socio-demographic and clinical characteristics. At enrollment, patient’s age, sex, level of education and occupation were recorded. Patients who met the inclusion criteria were conveniently recruited for this study. Outpatients with symptoms unrelated to UTI and those who were admitted were excluded.

### Urine sample collection and processing

Urine samples were collected according to the standard procedures as previously described [22]. For adult participants, the midstream urine method was used and the catch clean method was used for children. Participants were instructed to collect urine samples in two separate sterile containers. One bottle containing the sample was used for the dipstick urine test to detect leucocytes at the study health facility. The other container was immediately stored in the refrigerator (4 °C), where culture was done within 24 h after sample collection [22].

### Isolation of microorganisms

Urine samples were plated on cysteine lactose electrolytes deficient (CLED) agar medium. A loop calibrated to deliver approximately 0.001 mL was used to inoculate urine in CLED agar plates and incubated aerobically at 37^o^C for 24 h. The growth of one type of organism of > 10^5^ colony-forming units was considered bacteriuria [23, 24]. Clinical isolates were identified and confirmed biochemically using standard laboratory workflow [25, 26]. Confirmed bacteria isolates were suspended in nutrient broth, supplemented with 16% glycerol, and frozen at -80^o^C. The isolated bacteria samples were used for testing antimicrobial susceptibility and biofilm production.

### Antimicrobial susceptibility testing

Antimicrobial susceptibility tests were performed according to standards of Clinical and Laboratory Standards Institute (CLSI) guidelines using Kirby ‘Bauer’s disc diffusion method [27]. Briefly, bacterial suspensions in physiological saline solution were spread platted on Mueller-Hinton Agar. Antimicrobial-impregnated disks, selected based on CLSI, were then placed on the culture medium surface. Commercially available antibiotic sensitivity discs that are widely used in Tanzania, including ampicillin (10 µg), amoxicillin/clavulanic acid (20/10µg), ciprofloxacin (5 µg), co-trimoxazole (trimethoprim/ sulfamethoxazole) (1.25 µg/23.75), ceftriaxone (30 µg), and nitrofurantoin (300µg) were tested. For *P. aeruginosa*, additional antibiotic sensitivity discs, including imipenem (10 µg), amikacin (30 µg) and gentamicin (10 µg), were also tested. After incubating the plates at 37°C for 18–24 hours, the diameter (nearest whole mm) of the inhibition zones [27] for each antibiotic was measured. The interpretation breakpoints were based on whether the bacterium was susceptible (S), intermediate (I), or resistant (R) to the tested drugs according to the CLSI recommendations [27]. Antibiograms were validated using standardized control strains of *E. coli* (ATCC 25922), *S. aureus* (ATCC 25923) and *P. aeruginosa* (ATCC 27853).

### Determination of biofilm formation

To detect biofilm forming bacteria isolates, modified Congo red Agar method was used as previously described [28]. Briefly, bacteria strains were inoculated on blood base agar − 2 (BAB2) (40 gms/L BAB2, 10 gms/L glucose and 1000ml water), with 0.4 gms/L Congo red stain. At first, the Congo red stain was prepared as an aqueous solution and autoclaved (121 °C for 15 min) separately from the other medium constituents. The Congo red solution was added when the agar was cooled and incubated aerobically at 37 °C for 24 /48 hours. Black colonies with a dry crystalline consistency were interpreted as positive biofilm results. A red colored colony indicated negative biofilm results. *E. coli* ATCC 25,922 was used as positive control and *S. aureus* ATCC 25,932 as negative control for the CRA method.

### Statistical analysis

Statistical package for Social Sciences (SPSS) software (IBM, Armonk, NY, USA) was used to analyze the data. Descriptive statistics (median, IQR, mean + SD, percentages) were used for patients’ socio-demographic characteristics. Comparison between proportions for categorical variables in two independent groups was performed using the two-sided χ2 test or Fisher’s exact test. Cohen’s kappa coefficient was used to test the agreement of positive and negative UTIs between urine deep stick and bacteria culture methods. Multi-drug resistance (MDR) was defined as acquired non-susceptibility to at least one agent in three or more antimicrobial categories [29]. Binary logistic regression assessed the association between UTI and independent variables. A p-value < 0.05 was considered statistically significant.

### Ethical consideration

The study was approved by the Institutional review board of the Muhimbili University of Health and Allied Sciences (MUHAS) before the commencement of the study (DA.282/298/01.C/920). Permission to conduct the study was requested from the Morogoro regional referral hospital’s medical officer in charge. Written consent was obtained from all participants before enrollment.

## Results

### Socio-demographic characteristics of participants

A total of 344 patients attending the outpatient clinic presenting with UTI symptoms were recruited for this study from January to May 2022. Participants had a median age of 25.5 (1–83) years; the majority (63%) were young adults between 19 and 59 years old. About three-quarters of the participants (76.4%) were women. More than half of the female adults were pregnant (60%). Almost half of the adult participants (52.1%) had formal employment and three quarter (66.7%) were living with a partner. The socio-demographic characteristics of participants are shown in Table 1.

**Table 1:** Socio-demographic characteristics of participants

Category	Variable	Frequency n (%)
Age (n = 344)	Median age (range)	25.5 (1–83)
	Children (1–12 years)	63 (18.3)
	Adolescents (13–18)	31 (9.0)
	Young adults (19–59)	215 (62.5)
	Elders (≥60)	35 (10.2)
Sex (n = 344)	Male	81 (23.6)
	Female	263 (76.4)
Education Level (n = 250)	No formal education	7 (2.8)
	Primary	87 (34.8)
	Secondary	96 (38.4)
	Above secondary	60 (24.0)
Occupation (n = 165)	Formal employment	86 (52.1)
	Self employed	63 (38.2)
	Subsistence farmers	16 (9.7)
Pregnancy status (adult women) (n = 130)	YES	78 (60%)
	NO	52 (40%)
Marital status (n = 150)	Single	50 (33.3)
	Married	100 (66.7)

### Prevalence and determinants of UTI

The prevalence of UTI among patients attending the outpatient clinic presenting with UTI symptoms was 34.3% (118/344) and 41% (141/344) by dipstick urine analysis and culture, respectively. There was low agreement between dipstick urine analysis and culture in detecting positive UTI (Kappa = 0.11). The sensitivity and specificity of dipstick urine analysis were 43% and 69%, respectively, compared to the urine culture method.

The relationship between baseline parameters and the prevalence of UTI was evaluated. There was no significant difference in UTI prevalence between different levels of education (p = 0.13), marital status (p = 0.37) and occupation (p = 0.77). When comparing pregnant with non-pregnant adult women, there was no statistically discernible difference in the prevalence of UTI (p = 0.40). On logistic regression, elderly patients (≥ 60 years) had five times higher odds of having UTI as compared to adolescents. Female participants had almost two times higher odds of having UTI as compared to male participants at the borderline *p* = 0.05 (Table 2).

**Table 2:** Predictors of UTI

Variable	n (%)	Univariate analysis		Multivariate analysis	
		OR (95% CI)	p-value	aOR (95% CI)	p-value
Age (years)					
Children (1–12)	18 (28.6)	2.0 (0.8–5.2)	0.15	1.9 (0.7–4.9)	0.19
Young adults (19–59)	82 (38.1)	1.3 (0.6–2.9)	0.48	1.5 (0.7–3.3)	0.36
Elders (≥60)	16 (45.7)	4.5 (1.2–16.4)	**0.023**	4.8 (1.3–17.9)	**0.017**
Adolescents (13–18)	5 (16.1)	1		1	
**Sex**					
Female	90 (34.2)	1.6 (0.9–2.9)	0.09	1.9 (1.0-3.6)	**0.05**
Male	20 (24.7)	1		1	

### Antimicrobial susceptibility patterns

UPEC was the most common isolated bacteria, accounting for 47% (66/141) of all the isolated species. *P. aeruginosa, K. pneumoniae* and *P. mirabilis* contributed 17% (24/141), 11.4% (16/141) and 14.2% (20/141) of all isolated species, respectively. Other species, which were non-identified Gram negetive bacilli, accounted for 9.9% (14/141).

**Fig. 2:**
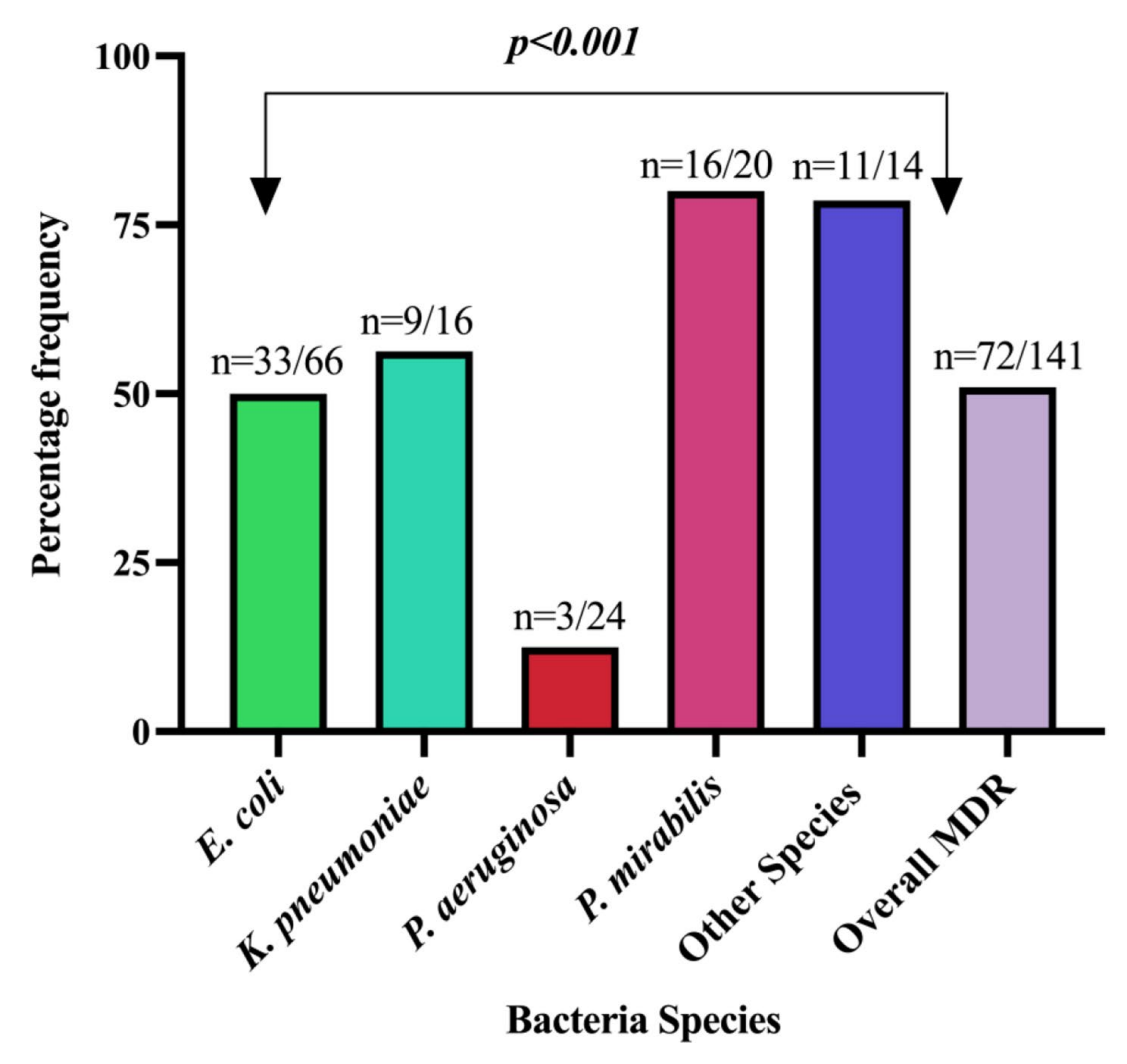
Proportion of MDR among the isolated bacteria species. Other species represent non-identified Gram negetive bacilli (*p-*value is based on the Chi-Square test)

A total of 72 (51%) of all isolated bacteria demonstrated resistance to at least one agent in three or more antimicrobial categories (MDR) Fig. 2. For *P. aeruginosa*, MDR was assessed in three antimicrobial families namely aminoglycosides (amikacin and gentamicin) flouroquinolones (ciprofloxacin) and carbapenems (imipenem). Isolates of *P. aeruginosa* displayed low rates of MDR, which differed significantly compared to other isolated bacteria (Fig. 2).

**Fig. 3:**
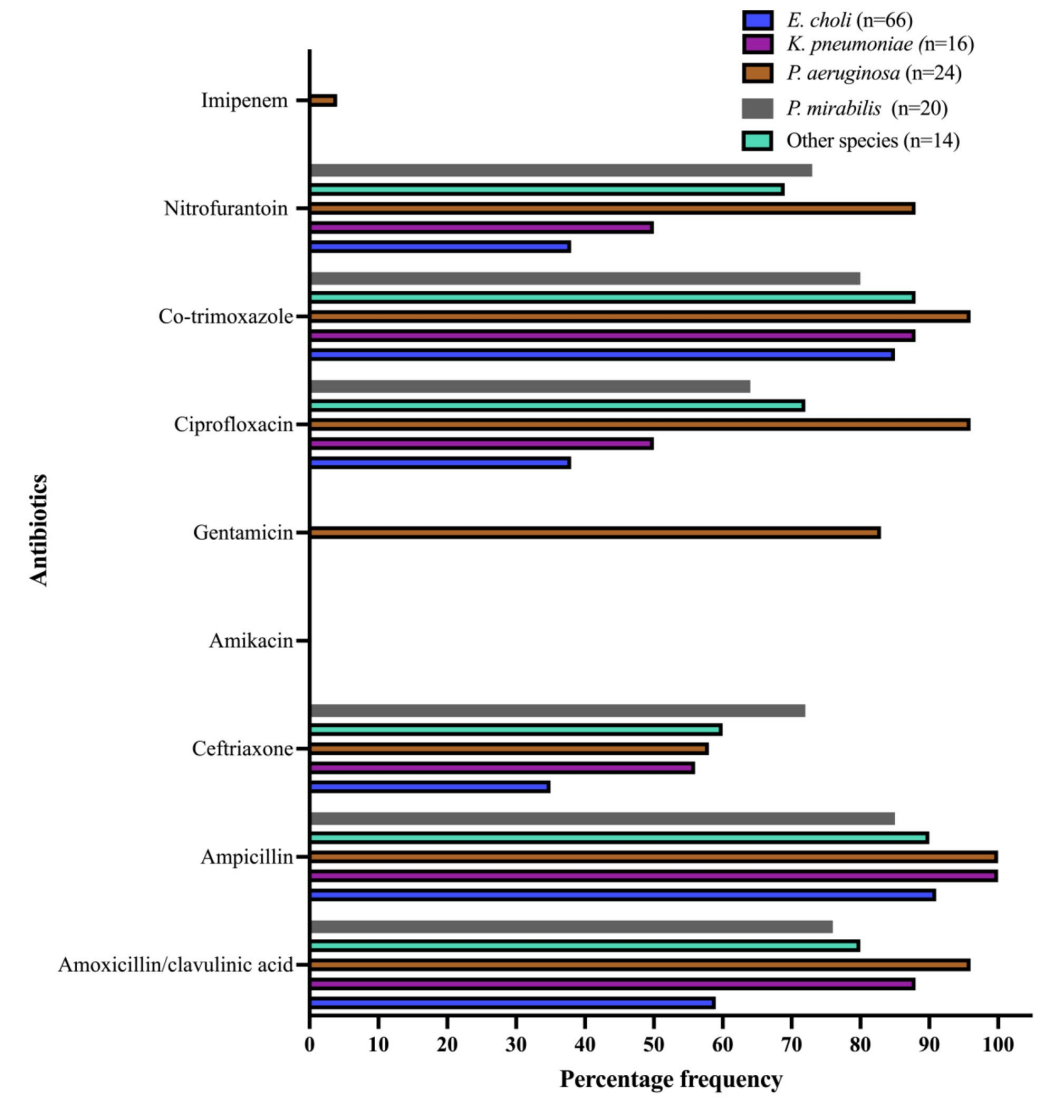
Antimicrobial resistance patterns among isolated bacteria (**`**n´ indicate the number of isolates under each bacteria specie)

Isolated species demonstrated moderate to low susceptibility to the tested antibiotics. *E. coli* showed fairly susceptibility against ciprofloxacin (59.1%), nitrofurantoin (57.6%) and ceftriaxone (50%). *P. aeruginosa* displayed the highest susceptibility against amikacin (87.5%) and imipenem (83%) and lower susceptibility against gentamicin. *K. pneumonia* demonstrated fair susceptibility against ciprofloxacin (50%). Figure 3 presents the AMR patterns for the isolated species.

### Biofilm formation

**Fig. 4:**
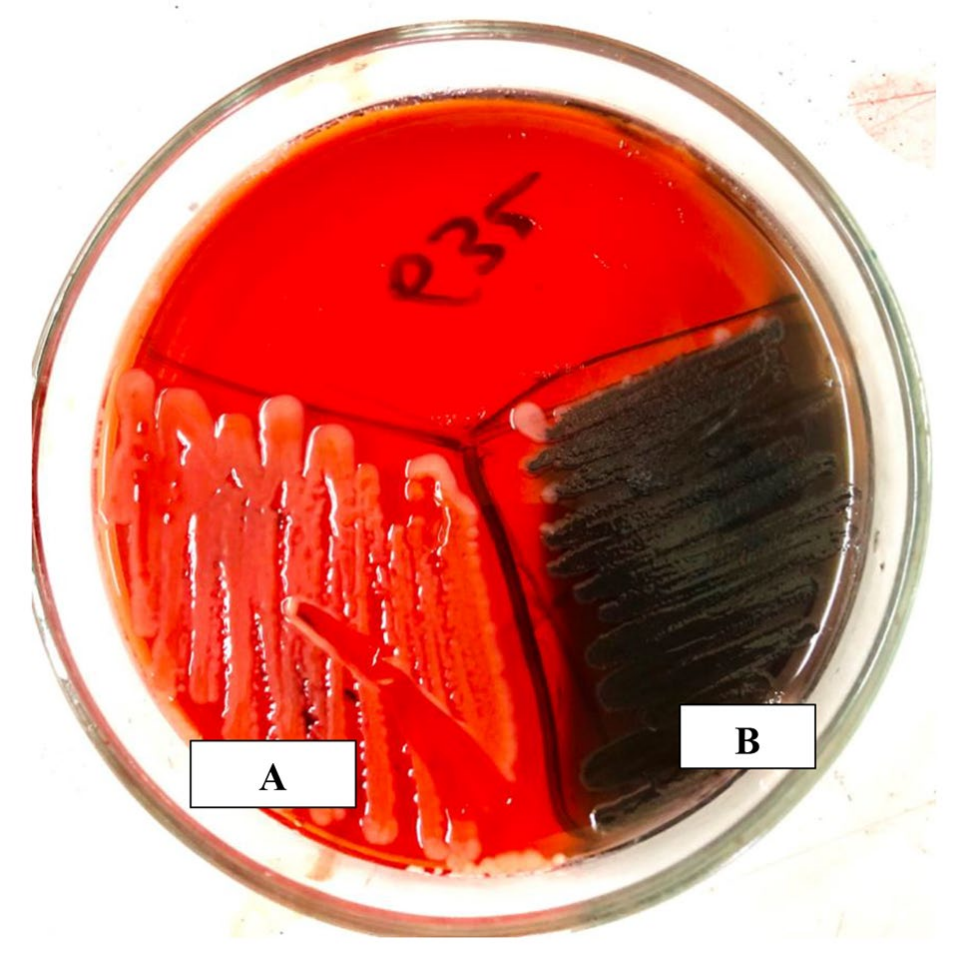
Congo Red Agar plates showing non-biofilm forming (A = red colored colonies) and biofilm forming (B = Dry crystalline Black colonies) UPEC isolates

The black colonies, with a dry crystalline consistency, were interpreted as positive biofilm results. In contrast, red colored colonies indicated negative biofilm results (Fig. 4). Of the 66 clinical isolates of *E. coli*, 34 (51.5%) formed biofilm on CRA.

More biofilm forming UPEC were MDR compared to UPEC non-biofilm forming isolates, although the difference was not statistically significant (p-value = 0.17). On the other hand, biofilm forming isolates demonstrated significantly higher resistance against co-trimoxazole and amoxycillin/clavulanic acid (p < 0.05), as shown in Table 3.

**Table 3:** Drug resistance among biofilm forming and non-biofilm forming UPEC isolates

Resistant to;	Biofilm forming *E. coli* n (%) N = 34	Non-biofilm forming *E. coli* n (%) N = 32	p-value
Amoxicillin/clavulanic acid	22 (69)	10 (31)	**0.013**
Ampicillin	27 (54)	23 (46)	0.30*
Ceftriaxone	12 (60)	8 (40)	0.18*
Ciprofloxacin	15 (65)	8 (35)	0.09
Co-trimoxazole	27 (59)	19 (41)	0.01*
Nitrofurantoin	13 (59)	9 (41)	0.11
MDR	20 (61)	13 (39)	0.17

*P-values are based on Fisher´s exact test

## Discussion

UTI is a public health problem accounting for more than 15% of all the antibiotics prescriptions among outpatients [30]. Infections forming biofilm are associated with AMR and recurrent UTIs [31, 32] both of which are currently increasing globally [4]. Nevertheless, data regarding the characterization of biofilm-forming UTIs in relation to AMR rates in sub-Saharan Africa are scanty. We report, a substantial proportion of outpatient clinic patients with UTI symptoms had UTI, high AMR rate to commonly used antibiotics and 50% of UPEC isolates forming biofilm UTI associated with antibiotics resistance. The study included an outpatient population presenting with UTI symptoms regardless of sex and age to reflect the real-world population distribution. To the best of our knowledge, this is the first study to report biofilm-forming patterns of UPEC and their correlation with antibiotics resistance from Tanzania.

In this study, 41% of patients attending the outpatient clinic with any UTI symptoms were found to have culture-positive UTI. This prevalence is higher than the global prevalence, which ranges from 13 to 33% but is comparable to that of other resource-limited countries [33]. Our finding is also relatively lower than that reported among children with symptoms attending pediatric clinics [34] and among women attending outpatient clinics (63%) [35] in Dar es Salaam, Tanzania. On the other hand, our finding is higher than that reported among symptomatic women in Mwanza, Tanzania (18%) [36] and among asymptomatic pregnant women in Arusha (31.6%) northern part of Tanzania [37]. Our results indicate the diverse burden of UTI across different geographical locations, which might explain the differences in social determinants of UTI.

This study found a higher prevalence of UTI in women than men at the borderline (p = 0.05). Usually, women have shorter urethra, which is close to the perineum, than men; thus, they are more prone to UTI than men [38]. The fact that there were fewer male participants in the current study may account for the lack of significant differences. In contrast, older people had a considerably higher chance of getting a UTI than teenagers, which could be attributed to immunosenescence, an age-related immunological shift [39]. Other baseline characteristics did not show a significant effect on UTI.

Isolated species exhibited moderate to high resistance patterns against commonly used antibiotics. Half of all isolated bacteria species were resistant to at least one agent in more than two classes of antibiotics, referred to as multi-drug resistance (MDR). All isolated species showed high resistance rates against co-trimoxazole and ampicillin, similar to other reported findings from previous studies [40], suggesting that the drugs might have limited susceptibility against UTI and should be less considered in the treatment regimen. *E. coli* was the most predominant isolated species and displayed moderate resistance rates against ceftriaxone (34%), ciprofloxacin (37%) and nitrofurantoin (37%) which is comparable to several other previous studies [41, 42]. On the contrary, previous studies reported a higher resistance rate against ceftriaxone, ciprofloxacin and nitrofurantoin [36] [43]. Ciprofloxacin and ceftriaxone are the drugs of choice for UTI in Tanzania, suggesting that a substantial proportion of UTIs from the setting may be susceptible to the first-line antibiotics. However, isolated species exhibited high resistance against amoxicillin/clavulanic acid, the drug of choice for UTI in pregnant and adolescent women [44], similar to a previous study from Uganda [45].

*P. aeruginosa* demonstrated the highest sensitivity rates (> 80%) against amikacin and imipenem with low rates of MDR. The reason for this finding could be that these are reserved antibiotics and not commonly used in health facilities thus, they have low chances for resistance development. On the contrary, *P. aeruginosa* showed a higher rate of resistance (85–100%) among the commonly used antibiotics corroborating the findings of previous studies [46]. The finding implies that most UTIs associated with *P. aeruginosa* may not respond to commonly prescribed antibiotics. Our findings contribute to the knowledge about antibiotic resistance patterns peculiar to this geographical location.

About half of isolated *E. coli* were biofilm formers on Congo Red Agar (CRA). This finding suggest that biofilm forming UTIs are common in Tanzania similar to the findings of previous studies from other regions [15–17]. The susceptibility pattern showed that biofilm-forming *E. coli* were more MDR (60%) compared to non-biofilm formers (40%). Although statistical significance was not reached for the overall MDR, biofilm formers showed significantly higher resistance rates against ciprofloxacin and co-trimoxazole as compared to non-biofilm formers. Previous studies from other regions reported significant association between biofilm forming capability and AMR for the same antibiotics as the present study [15–17]. One reason to explain this might be the difficult penetration of antimicrobials to the deeper layers of the biofilm matrix which expose bacteria to sub-therapeutic antimicrobial concentrations leading to resistance [12]. This finding informs the policy makers on the importance of considering the impact of biofilm infections in policies against antimicrobial resistnance.

The observed high antibiotic resistance patterns might be explained by the community´s inappropriate and overuse of antibiotics [47]. Miserably, AMR causes a stall in achieving the global sustainable development goal number 3.3, which aims at reducing the burden of infectious diseases by the end of 2030 [48]. The findings of this study underscores the need for strenghthening antimicrobial stewardship in health facilities [49].

UTI is treated empirically in Tanzania and other resource-limited countries. In the majority of primary healthcare facilities, urine analysis is based on dipstick urine analysis, which in this study was found to have a lower sensitivity (45%) than urine culture. This suggests that a method used in the standard of care for diagnosing UTIs may miss a considerable portion of individuals who have severe bacteriuria. The findings underscore the need for integrating urine culture and sensitivity to properly manage UTI in our health facilities.

### Conclusion and recommendations

We report a high prevalence of culture-positiveUTI among outpatients presenting with UTI symptoms from the study population. High prevalence rates of antibiotic resistance were also observed in clinical bacterial isolates. Slightly more than half of the isolated *E. coli* bacteria were biofilm forming, demonstrating higher antibiotic resistance to co-trimoxazole and amoxicillin/clavulanic acid than non-biofilm forming counterparts. A need to strengthen AMR stewardship programs in health facilities is urgently advocated in order to halt the spread of AMR.

## Data Availability

All data generated or analyzed during this study are included in this manuscript.

## Abbreviations

CLSI: Clinical and laboratory standards institute
MDR: Multi drug resistance
SOPs: Standard Operating procedures
UPEC: Uropathogenic *E. coli*
MUHAS: Muhimbili University of Health and Allied Sciences
